# Diverse genotypes of human enteric and non-enteric adenoviruses circulating in children hospitalized with acute gastroenteritis in Thailand, from 2018 to 2021

**DOI:** 10.1128/spectrum.01173-23

**Published:** 2023-08-17

**Authors:** Arpaporn Yodmeeklin, Kattareeya Kumthip, Nuthapong Ukarapol, Hiroshi Ushijima, Niwat Maneekarn, Pattara Khamrin

**Affiliations:** 1 Department of Microbiology, Faculty of Medicine, Chiang Mai University, Chiang Mai, Thailand; 2 Center of Excellence in Emerging and Re-emerging Diarrheal Viruses, Chiang Mai University, Chiang Mai, Thailand; 3 Department of Pediatrics, Faculty of Medicine, Chiang Mai University, Chiang Mai, Thailand; 4 Division of Microbiology, Department of Pathology and Microbiology, Nihon University School of Medicine, Tokyo, Japan; National Chung Hsing University, Taichung, Taiwan

**Keywords:** adenovirus, children, gastroenteritis, Thailand

## Abstract

Human adenovirus (HAdV) is a common viral pathogen that causes diarrhea in children worldwide. The aim of this study was to investigate the prevalence and genotype diversity of HAdV strains circulating in children admitted to the hospitals with acute gastroenteritis (AGE) in Chiang Mai, Thailand, from 2018 to 2021. A total of 1,790 stool samples were screened for HAdV by PCR method, and 80 (4.5%) were positive for HAdV. Of these, children under 5 years of age accounted for 90.0% of HAdV-positive cases with the highest infection rate at the age group of 48–60 months old. The infection rate was not significantly different between boys and girls. The HAdV infection was detected sporadically throughout the year without a discrete seasonal pattern. Five species of both enteric and non-enteric HAdVs (A, B, C, E, and F) with 10 different genotypes, including HAdV-F41 (25.0%), HAdV-B3 (17.5%), HAdV-F40 (16.3%), HAdV-C1 (15.0%), HAdV-C5 (7.5%), HAdV-C2 (6.3%), HAdV-B7 (5.0%), HAdV-A12 (3.8%), HAdV-E4 (2.5%), and HAdV-B11 (1.3%), were detected in this study. In conclusion, our study reported the prevalence and seasonality of HAdV infection with a wide variety of HAdV genotypes circulating in children hospitalized with AGE during a period of 2018–2021 in Chiang Mai, Thailand.

**IMPORTANCE:**

In the present study, the prevalence of human adenovirus (HAdV) infection in children with acute gastroenteritis (AGE) in Chiang Mai, Thailand, from 2018 to 2021 was detected at 4.5%. Diverse species and genotypes of HAdVs (HAdV-A12, HAdV-B3, HAdV-B7, HAdV-B11, HAdV-C1, HAdV-C2, HAdV-C5, HAdV-E4, HAdV-F40, and HAdV-F41) had been identified. The highest infection rate was found in children aged 48–60 months old. The HAdV infection was detected sporadically throughout the year. These findings imply that a wide variety of HAdV genotypes circulate in pediatric patients with AGE in Chiang Mai, Thailand.

## INTRODUCTION

Acute gastroenteritis (AGE) is one of the major causes of morbidity and mortality in infants and young children worldwide, especially in low- and middle-income countries. More than 70% of all diarrheal episodes are induced by gastroenteritis viruses (1). Human adenovirus (HAdV) has also been recognized as an important virus that causes diarrhea in pediatric patients (2, 3). HAdV can infect humans of all age groups, but it is most commonly observed in the pediatric population, particularly in infants and young children. The HAdV infection can result in a wide range of symptoms, varying from mild to severe, and in some cases, it can even be fatal (4). HAdV belongs to the genus *Mastadenovirus* in the family *Adenoviridae*. It is a non-enveloped virus containing a linear double‐stranded DNA genome with a genomic size of about 34–36 kbp (5). The three major capsid genes (penton, hexon, and fiber) are identified as hotspots for homologous recombination, serving as one major factor in HAdV genome diversity and viral evolution. The recombination sites are located around the border of hypervariable loops 1 and 2 within the hexon gene. These loops are essential for the formation of type-specific epitopes, which are recognized by neutralizing antibodies and play a crucial role in HAdV evolution (6 – 8). In addition, the hexon gene of HAdV has been reported to contain several hypervariable regions (HVR1–HVR7), and type-specific epitopes are located in one or more HVRs of the hexon protein (9). The HVRs of hexon gene are dissimilar in different genotypes and, therefore, are commonly used as a target for the type-specific primers designed for the detection and genotyping of HAdV (10 – 13). Currently, 10 species (A–J) and more than 100 genotypes of HAdV have been identified (14). HAdVs are associated with a wide spectrum of symptoms similar to the common cold, including rhinorrhea, fever, cough, and sore throat. Lower respiratory tract symptoms such as bronchitis, bronchiolitis, and pneumonia can be severe and even fatal. Other diseases such as gastroenteritis, conjunctivitis, cystitis, myocarditis, cardiomyopathy, and meningoencephalitis can also be associated with adenovirus infections (4). HAdV diarrhea can be persistent and severe in immunosuppressed and immunocompromised patients (15, 16). Among the HAdV species, enteric HAdV species F (genotypes 40 and 41) is associated with acute gastroenteritis in humans and is responsible for up to 1.5%–30% of diarrheal cases worldwide (17 – 21). However, non-enteric adenovirus species, such as HAdV-A (genotypes 12, 18, 31), HAdV-B (genotypes 3, 7, 11, 16, 21), HAdV-C (types 1, 2, 5, 6), HAdV-D (genotypes 10, 28, 29, 30, 32, 37, 61, 64, 70), and HAdV-G (genotypes 52), have also been reported to associate with gastroenteritis (19, 22 – 26).

Epidemiological studies of HAdV infection in diarrheal cases have been reported from several countries around the world, including Australia (27), the United States (16, 28, 29), Switzerland (30), and Italy (31) with the prevalence ranging from 6.0% to 26.0%. Additionally, HAdV infection has also been reported in several countries in Asia, including Japan (32, 33), Korea (34), China (35, 36), and Thailand (19), with the prevalence ranging from 1.0% to 13.5%. Not only enteric HAdV species F (genotypes 40 and 41) frequently identified in stool samples from patients with AGE but also non-enteric HAdVs, such as HAdV-A, HAdV-B, HAdV-C, HAdV-D, and HAdV-G, are also associated with AGE infections (19, 22 – 26). In Thailand, epidemiology of HAdV infection in patients with acute gastroenteritis has been reported from Lopburi province since 2006–2007 with the prevalence of 1.5% (37) and from Bangkok and Khon Kaen provinces in 2009–2012 with the prevalence of 5.8% (38). In addition, epidemiology of HAdV infection has also been reported from Chiang Mai province in 2007 with the prevalence of 0.6% (39) and in 2011–2017 with the prevalence of 7.2% (19). The aim of the present study was to investigate the prevalence and to characterize the HAdV genotypes circulating in pediatric patients admitted to the hospitals with acute gastroenteritis in Chiang Mai, Thailand, during a period of 4 years from 2018 to 2021.

## RESULTS

### Detection rate, distribution of HAdV genotypes in different age groups and genders, and co-infections

HAdVs were detected in 80 out of 1,790 (4.5%) fecal specimens collected from patients with AGE during the 4-year study period (January 2018–December 2021) (Table 1). The yearly prevalences were 6.2% (45/728), 3.9% (22/563), 2.3% (4/175), and 2.8% (9/324) in 2018, 2019, 2020, and 2021, respectively. The majority of HAdV infection (72/80; 90.0%) was observed in children under 5 years of age. Among 80 HAdV-infected patients, 39 (48.8%) and 41 (51.2%) were boys and girls, respectively. The difference of infection rate between boys and girls was not statistically significant (*P* = 0.111). However, considering the distribution of HAdV genotype infection in different genders, HAdV-B11 and HAdV-E4 were found to infect solely in boys and girls, respectively. In addition, HAdV-A12, HAdV-B7, HAdV-C1, HAdV-C5, and HAdV-F40 were found to infect the boys with higher infection rates ranging from 58.3% to 75.0%. On the contrary, HAdV-B3, HAdV-C2, and HAdV-F41 were found to infect the girls with higher infection rates ranging from 60.0% to 65.0%

**TABLE 1:** Human adenovirus infection in children with acute gastroenteritis in accordance with the age and gender during 2018–2021

Age groups (months)	2018			2019			2020			2021			No. of total positive/ tested samples 2018–2021 (%)
	No. of positive/tested samples (%)	Positive male (%)	Positive female (%)	No. of positive/tested samples (%)	Positive male (%)	Positive female (%)	No. of positive/tested samples (%)	Positive male (%)	Positive female (%)	No. of positive/tested samples (%)	Positive male (%)	Positive female (%)	
<6	2/70 (2.9)	1 (1.4)	1 (1.4)	0/94 (0)	0 (0)	0 (0)	1/30 (3.3)	0 (0)	1 (3.3)	0/72 (0)	0 (0)	0 (0)	3/266 (1.1)
6 to <12	7/124 (5.6)	2 (1.6)	5 (4.0)	4/87 (4.6)	2 (2.3)	2 (2.3)	0/37 (0)	0 (0)	0 (0)	3/61 (4.9)	2 (3.3)	1 (1.6)	14/309 (4.5)
12 to <24	14/242 (5.8)	8 (3.3)	6 (2.5)	9/155 (5.8)	3 (1.9)	6 (3.9)	2/40 (5)	0 (0)	2 (5)	5/108 (4.6)	3 (2.8)	2 (1.9)	30/545 (5.5)
24 to <36	6/130 (4.6)	3 (2.3)	3 (2.3)	2/89 (2.2)	2 (2.2)	0 (0)	0/19 (0)	0 (0)	0 (0)	1/19 (5.3)	0 (0)	1 (5.3)	9/257 (3.5)
36 to <48	5/57 (8.8)	2 (3.5)	3 (5.3)	3/55 (5.5)	1 (1.8)	2 (3.6)	0/17 (0)	0 (0)	0 (0)	0/11 (0)	0 (0)	0 (0)	8/140 (5.7)
48 to <60	4/37 (10.8)	3 (8.1)	1 (2.7)	3/38 (7.9)	3 (7.9)	0 (0)	1/13 (7.7)	0 (0)	1 (7.7)	0/18 (0)	0 (0)	0 (0)	8/106 (7.5)
60 to <180	7/68 (10.3)	4 (5.9)	3 (4.4)	1/45 (2.2)	0 (0)	1 (2.2)	0/19 (0)	0 (0)	0 (0)	0/35 (0)	0 (0)	0 (0)	8/167 (4.8)
Total	45/728 (6.2)	23 (3.2)	22 (3.0)	22/563 (3.9)	11 (2.0)	11 (2.0)	4/175 (2.3)	0 (0)	4 (2.3)	9/324 (2.8)	5 (1.5)	4 (1.2)	80/1,790 (4.5)

Considering the HAdV infection in different age groups, the highest infection rate was observed at the age group of 48 to <60 months (7.5%), followed by 36 to <48 months (5.7%), 12 to <24 months (5.5%), 60 to <180 months (4.8%), 6 to <12 months (4.5%), 24 to <36 months (3.5%), and <6 months (1.1%). However, the infection rate in children with different age groups was not statistically different (*P* = 0.067). The median of HAdV infection rate in children with different age groups was 4.8% with an interquartile of 2.2. Among HAdV-positive cases, sole infection by HAdV was observed at 67.5% (54/80) and co-infection with other enteric viruses was detected at 32.5% (26/80) (Table 2). Co-infection of HAdV with norovirus (NoV) or rotavirus (RV) was commonly detected at the prevalence of 30.8% (8/26) and 26.9% (7/26), respectively. Co-infection of HAdV with bocavirus (BoV) was detected at 7.7% (2/26), while one each of the sample was co-infected with sapovirus (SaV), Aichivirus (AiV), human parechovirus (HPeV), or saffold virus (SAFV) at the prevalence of 3.8% (1 each of 26). Mixed infections of HAdV with two different other gastroenteritis viruses were found in 4 of 26 cases (15.4%), while mixed infections with three different other viruses were observed in 1 of 26 cases (3.8%).

**TABLE 2:** Detection rate of human adenovirus infection and co-infections with other gastroenteritis viruses in children with acute gastroenteritis in Thailand from 2018 to 2021^*b*^

Years	No. of specimens tested	No. of HAdV positive (%)	No. of HAdV single infection (%)	No. of HAdV co-infection (%)	No. and patterns of HAdV co-infection											
					RV	NoV	SaV	AiV	BoV	HPeV	SAFV	≥2 Viruses^*a*^				
												RV + NoV	RV + BoV	NoV + EV	EV + BoV	RV + AiV + EV
2018	728	45 (6.2)	27 (60.0)	18 (40.0)	6	4	1	1	–^*c*^	1	1	1	–	1	1	1
2019	563	22 (3.9)	16 (72.7)	6 (27.3)	–	3	–	–	2	–	–	–	1	–	–	–
2020	175	4 (2.3)	3 (75.0)	1 (25.0)	1	–	–	–	–	–	–	–	–	–	–	–
2021	324	9 (2.8)	8 (88.9)	1 (11.1)	–	1	–	–	–	–	–	–	–	–	–	–
Total	1,790	80 (4.5)	54 (67.5)	26 (32.5)	7	8	1	1	2	1	1	1	1	1	1	1

^*a*^ HAdV co-infection with other two or more enteric viruses.

^*b*^ RV, rotavirus; NoV, norovirus; SaV, sapovirus; AiV, Aichivirus; BoV, bocavirus; HPeV, human parechovirus; SAFV, saffold virus; EV, enterovirus.

^*c*^ –, viruses were not detected.

### Monthly distribution of HAdV infection

**Fig 1:**
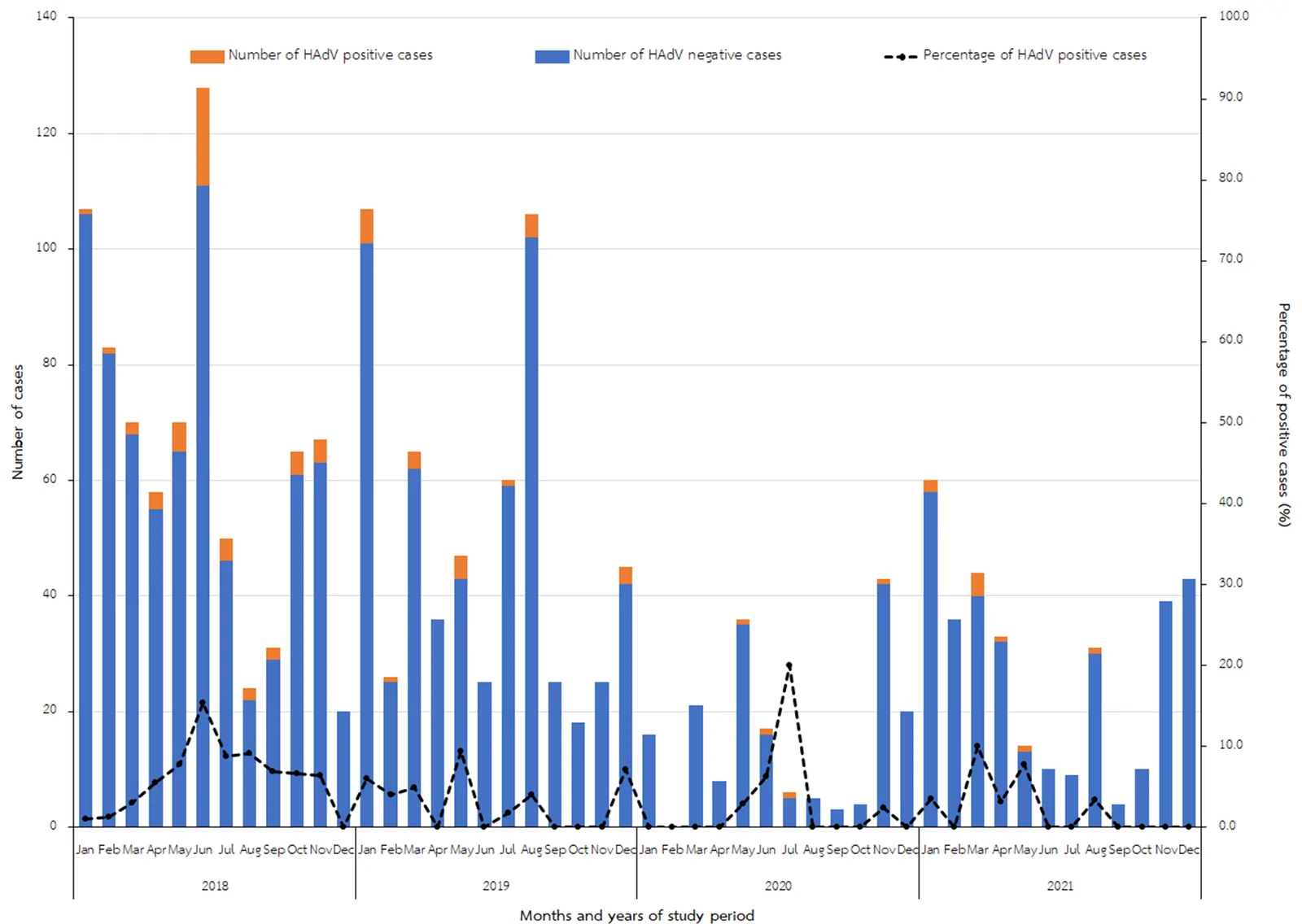
Monthly distribution of human adenovirus infection in children with acute gastroenteritis during 2018–2021. The left axis indicates the number of cases, and the right axis indicates the percentage of positive cases (%). The length of the blue bar indicates the number of cases tested, the length of the orange bar indicates the number of HAdV-positive cases, and the dot color indicates the percentage of HAdV-positive cases.

The seasonality of HAdV infection is shown in Fig. 1. In this study, HAdV was detected sporadically all year round with a slightly higher in the rainy season in Thailand (May–July) (*P* = 0.005). The highest monthly distribution of HAdV infection rate varied year by year, i.e., 15.3% in June of 2018, 9.3% in May of 2019, 20.0% in July of 2020, and 10.0% in March of 2021. However, it should be noted that the total number of fecal specimens collected in 2020 was remarkably lower than those of the other years due to the pandemic of SARS-CoV-2, which affected the number of diarrheic patients hospitalized in 2020.

### HAdV genotypes and phylogenetic analysis

**Fig 2:**
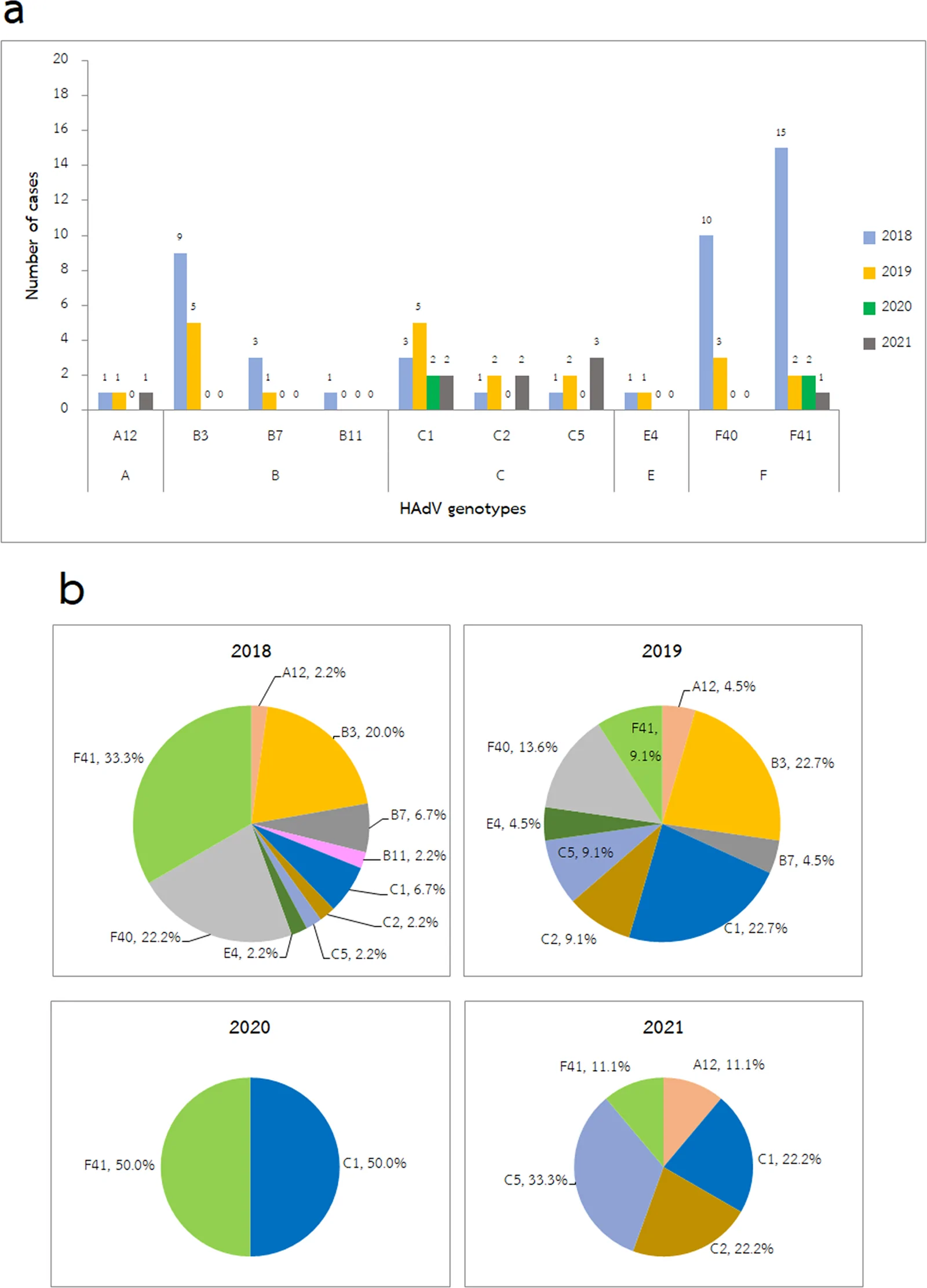
Distribution of human adenovirus genotypes in patients with acute gastroenteritis during 2018–2021. (**a**) Bar chart indicates the number of HAdV genotypes; the *x*-axis indicates the HAdV genotypes detected, and the *y*-axis indicates the number of cases. (**b**) Pie charts indicate the percentage of HAdV genotypes detected in each year during the study period of 2018–2021.

HAdV genotypes and phylogenetics of 80 HAdV strains detected in this study were analyzed based on the nucleotide sequence of the partial hexon gene. It was found that five species (A, B, C, E, and F) of HAdV were detected during the study period of 2018–2021 and wide varieties of HAdV genotypes were identified. Overall, the HAdV-F41 was detected as the most predominant genotype (25.0%; 20/80), followed by HAdV-B3 (17.5%; 14/80), HAdV-F40 (16.3%; 13/80), HAdV-C1 (15.0%; 12/80), HAdV-C5 (7.5%; 6/80), HAdV-C2 (6.3%; 5/80), HAdV-B7 (5.0%; 4/80), HAdV-A12 (3.8%; 3/80), HAdV-E4 (2.5%; 2/80), and HAdV-B11 (1.3%; 1/80) (Fig. 2). In fact, the predominant genotype had changed year by year, for example, HAdV-F41 and HAdV-F40 were detected as the most predominant genotypes in 2018, whereas in 2019, they were replaced by HAdV-B3 and HAdV-C1 genotypes. In 2020, only two genotypes, HAdV-C1 and HAdV-F41, were detected. In 2021, various HAdV genotypes were detected and HAdV-C5 was the most predominant genotype.

**Fig 3:**
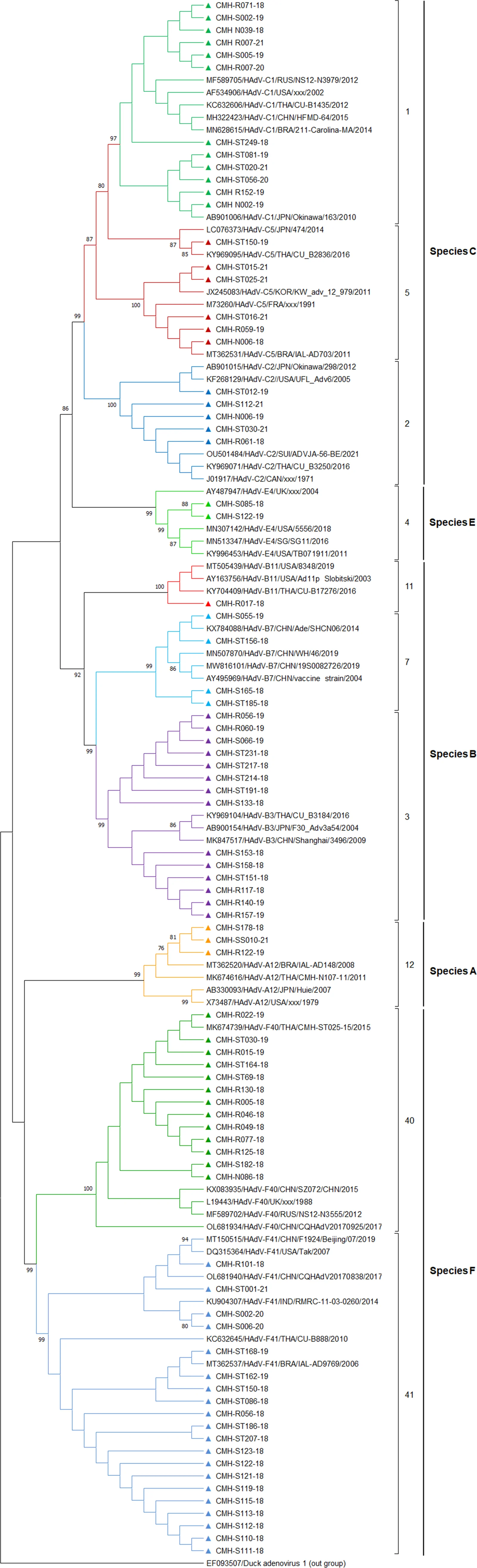
Phylogenetic analysis of partial nucleotide sequences (443 bp) of the hexon gene. The phylogenetic tree of human adenovirus strains detected in Thailand during 2018–2021 (80 sequences) and reference strains available in the Genbank database was constructed by MEGA X software with GTR + G + I model and supported by 1,000 bootstraps. The triangle indicates the HAdV strains detected in this study, and HAdVs with different genotypes are indicated by different colors. The reference strains of each HAdV genotype are indicated by strain name and accession number.

Phylogenetic analysis revealed that five species (A, B, C, E, and F) of HAdVs were detected in this study (Fig. 3). Three strains of HAdV-A12 were closely related to the reference strains detected previously in Thailand, Japan, Brazil, and the United States with the nucleotide (nt) sequence identities ranging from 97.2% to 98.8%. The HAdV-B3 (14 strains) showed 99.3%–99.5% nt sequence identities with HAdV-B3 reference strains reported previously from Thailand, Japan, and China. Four strains of HAdV-B7 showed a high degree of nt sequence similarities (99.3%–100%) with those of the reference strains reported previously from China. One strain of HAdV-B11 had 100% nt sequence identity with the reference strains detected previously in Thailand and the United States. The HAdV species C detected in this study showed highly similar to the reference strains previously reported worldwide. Of these, 12 strains of HAdV-C1 showed 97.9%–99.0% nt sequence identities with HAdV-C1 reported from Thailand, China, Japan, Russia, Brazil, and the United States, while 5 strains of HAdV-C2 were closely related (98.1%–99.0%) to the reference strains detected in Thailand, China, Japan, Switzerland, and the United States. Six strains of HAdV-C5 were closely related to the HAdV-C5 reference strains reported previously from Thailand, Japan, Korea, Brazil, and France with the nt sequence identities ranging from 93.2% to 100%. In addition, two strains of HAdV-E4 were closely related (96.1%–99.5% nt sequence identities) to the HAdV-E4 reference strains detected previously in Singapore, the United States, and the United Kingdom. Phylogenetic analysis of species F, HAdV-F40 (13 strains) and HAdV-F41 (20 strains), showed that they were closely related to the reference strains reported previously from Thailand, China, Russia, the United States, India, and Brazil with the nt sequence identities ranging from 98.1% to 100% and 97.7% to 100%, respectively.

## DISCUSSION

Epidemiology of HAdV infection in children with acute gastroenteritis in Thailand has been reported sporadically since 2006–2007 (37, 39). The prevalence of HAdV infection in Thailand ranged from 1.5% in Lopburi province in 2006–2007 (37) to 5.8% in Bangkok and Khon Kaen in 2009–2012 (38). In Chiang Mai province, the prevalence of HAdV infection was reported initially in 2007 at 0.6% (39), and the prevalence had increased markedly to 7.2% (ranged 3.6%–11.6%) during the study period of 2011–2017 (19). The present study is a follow-up study of HAdV infection in children with acute gastroenteritis in Chiang Mai province during a period of 2018–2021 which revealed an overall prevalence at 4.5% (ranged 1.1%–7.5%) (Table 1). The HAdV infection rate of 2018–2021 in this study is significantly lower than those of our previous study conducted in the same geographical area in 2011–2017 (19) with a *P*-value of <0.00048. It should be noted that in 2020 and 2021 during the pandemic of COVID-19 where the stool samples were collected in a much smaller number than those of the other study years, the HAdV infection rates were 2.3% and 2.8%, respectively (Table 1). The growing evidence suggests that the implementation of control measures for COVID-19, such as social distancing, mask-wearing, and increased hand hygiene, has proven effective to reduce COVID-19 infection along with many other infectious diseases, including acute gastroenteritis in children (40 – 42). The small number of acute gastroenteritis cases collected in 2020 and 2021 with low HAdV infection rates might be, at least in part, due to the influence of control measures for COVID-19. It should be pointed out that five species (A, B, C, E, and F) of HAdV were detected in this study and both enteric HAdV (species F) and non-enteric HAdV (species A, B, C, and E) were detected in patients with acute gastroenteritis (Fig. 3). However, in general, the enteric HAdV-F41 (25.0%) and HAdV-F40 (16.3%) were predominantly circulating in pediatric patients with acute gastroenteritis (Fig. 2). The results are more or less the same as our previous study conducted during 2011–2017 in the same geographical area where the enteric HAdV-F41 was the most predominant genotype with the prevalence of 22.4% (19). The molecular detection and genotyping of HAdV are commonly based on the analysis of nucleotide and amino acid sequences of the hexon gene (10 – 13). Even though genotype-specific primers targeting the hexon gene can be used successfully in the multiplex-PCR method for the detection and genotyping of HAdV in this study and in other studies (10, 19), it has a limitation to be used for genotyping of HAdV recombinant strain. Homologous recombination is the main driver of genetic diversity and evolution of HAdV, especially the recombination between the hexon, penton base, and fiber genes (5, 43). Conceivably, identification of HAdV genotype based only on the hexon gene sequence may lead to a wrong conclusion of HAdV genotype if it is the recombinant strain. For accurate identification of HAdV recombinant strain, analysis of whole-genome sequence encompassing penton base, hexon, and fiber genes is required (5, 43 – 45).

In addition, HAdV infections detected in this study were co-infected with many other enteric viruses, including rotavirus, norovirus, sapovirus, Aichivirus, bocavirus, human parechovirus, saffold virus, and enterovirus at 32.5% (Table 1). Co-infections by other enteric viruses are commonly reported elsewhere, such as Turkey (46), Egypt (47), Brazil (48), the United States (49), Bangladesh (25), and France (50) which demonstrated the co-infection of HAdV with rotavirus, norovirus, astrovirus, enterovirus, human parechovirus A, sapovirus ranging from 2.0% to 22.0%. This observation is similar to our previous studies conducted in the same geographical area during 2011–2017 (19). The remaining 67.5% of HAdV-positive cases were solely infected with HAdV, suggesting the etiologic role of HAdV in acute gastroenteritis. Nevertheless, some diarrheic cases may be associated with bacteria or other pathogens, which have not been investigated in this study, and could not be ruled out.

In conclusion, this study reported the prevalence and diversity of HAdV genotypes circulating in pediatric patients hospitalized with acute gastroenteritis, especially in children under 5 years of age (90.0%). Phylogenetic analysis revealed extensively diverse species and genotypes of HAdV, not only enteric HAdV species F (HAdV-F40 and HAdV-F41) but also other non-enteric HAdVs, including species A, B, C, and E have been reported in this study, and HAdV-F41 is the most predominant genotype. Molecular epidemiological surveillance of HAdV is essential to be conducted continually in several countries in order to obtain information from different countries worldwide. The information of the HAdV species and genotypes circulating in children with acute gastroenteritis in different countries worldwide is useful for the development of effective vaccine to reduce the burden of diarrheal disease associated with HAdV infection in the future.

## MATERIALS AND METHODS

### Patients and specimen collection

A total of 1,790 fecal specimens were collected from children hospitalized with acute gastroenteritis from five hospitals (Maharaj Nakorn Chiang Mai Hospital, Nakornping Hospital, Sanpatong Hospital, Rajavej Chiang Mai Hospital, and Sansai Hospital) in Chiang Mai, Thailand, between January 2018 and December 2021. The inclusion criteria of the patients in this study were the inpatients who were suffering from acute gastroenteritis with the symptoms including nausea, vomiting, abdominal pain, and diarrhea. The patients had sudden passages of loose or watery stools more than three times per day with the exclusion of bloody stools. The age of the patients ranged from neonate up to 15 years of age. All fecal samples were stored at −20°C until use. The study was conducted with the approval of the Institutional Ethics Committee of the Faculty of Medicine, Chiang Mai University (MIC-2557-02710).

### Sample preparation

Adenovirus dsDNA was extracted from the supernatant of a 10% fecal suspension in phosphate-buffered saline (pH 7.4) using a Geneaid Viral Nucleic Acid Extraction Kit II (Geneaid, Taipei, Taiwan) according to the manufacturer’s protocol. The viral genomic DNA was either subjected immediately to PCR assay or stored at −70°C until use.

### Detection of HAdV

HAdV genome was detected by PCR using the forward primer Ad1 (5′-TTCCCCATGGCTCAYAACAC-3′) in combination with the reverse primer Ad2 (5′-CCCTGGTAKCCRATRTTGTA-3′) which specifically amplified the hexon gene to generate a PCR product size of 482 bp as described previously (10). The reaction was performed under the following thermal cycling conditions: 94°C for 3 min, 35 cycles of 94°C for 1 min, 50°C for 1 min, 72°C for 1 min, and a final extension step at 72°C for 10 min, using a thermal cycler (SimpliAmp, Life Technologies Holdings Pte Ltd, Singapore). In addition to the screening of HAdV, all samples were also tested for the presence of several other diarrheal viruses, including RV, NoV, SaV, AiV, BoV, HPeV, SAFV, and EV using RT-PCR methods, as described previously (51) in order to investigate a situation of co-infections of HAdV by several other diarrheal viruses.

### Phylogenetic analysis

The PCR products were purified using a Gel/PCR DNA Fragments Extraction Kit (Geneaid, Taipei, Taiwan) according to the manufacturer’s protocol. The purified PCR products were then sequenced by a fluorescence-based cycle sequencing method (First Base Laboratories Sdn Bhd Selangor Darul Ehsan, Malaysia). The obtained nucleotide sequences of HAdV detected in this study were analyzed in comparison with those of the reference strains available in the NCBI GenBank database. Phylogenetic analysis of the partial hexon gene was performed using MEGA X software based on the Maximum Likelihood method and General Time Reversible model (52). Statistical analysis was performed using the bootstrapping method with 1,000 replicates.

### Statistical analysis

Statistical analysis was performed using SPSS version 16.0 software (SPSS Inc, Chicago, IL, United States). Statistical differences were determined using the Chi-square test, and *P* values <0.05 were considered a statistically significant difference.

## Data Availability

The nucleotide sequences of HAdV described in this study are available in the GenBank database under the accession numbers OQ513376 to OQ513455.

## References

[1] Babaei A , Rafiee N , Taheri B , Sohrabi H , Mokhtarzadeh A . 2022. Recent advances in early diagnosis of viruses associated with gastroenteritis by biosensors. Biosensors 12:499. doi:10.3390/bios12070499 35884302 PMC9313180

[2] Gasparinho C , Mirante MC , Centeno-Lima S , Istrate C , Mayer AC , Tavira L , Nery SV , Brito M . 2016. Etiology of diarrhea in children younger than 5 years attending the bengo general hospital in Angola. Pediatr Infect Dis J 35:e28–34. doi:10.1097/INF.0000000000000957 26761347

[3] Stuempfig ND , Seroy J . 2022. Viral gastroenteritis. In Statpearls. Treasure Island (FL): StatPearls Publishing [Updated 2022 Jun 21].

[4] Shieh W-J . 2022. Human adenovirus infections in pediatric population - an update on clinico-pathologic correlation. Biomed J 45:38–49. doi:10.1016/j.bj.2021.08.009 34506970 PMC9133246

[5] Dhingra A , Hage E , Ganzenmueller T , Böttcher S , Hofmann J , Hamprecht K , Obermeier P , Rath B , Hausmann F , Dobner T , Heim A . 2019. Molecular evolution of human adenovirus (HAdV) species C. Sci Rep 9:1039. doi:10.1038/s41598-018-37249-4 30705303 PMC6355881

[6] Matsushima Y , Shimizu H , Phan TG , Ushijima H . 2011. Genomic characterization of a novel human adenovirus type 31 recombinant in the hexon gene. J Gen Virol 92:2770–2775. doi:10.1099/vir.0.034744-0 21880842

[7] Bruder JT , Semenova E , Chen P , Limbach K , Patterson NB , Stefaniak ME , Konovalova S , Thomas C , Hamilton M , King CR , Richie TL , Doolan DL . 2012. Modification of Ad5 hexon hypervariable regions circumvents pre-existing Ad5 neutralizing antibodies and induces protective immune responses. PLoS One 7:e33920. doi:10.1371/journal.pone.0033920 22496772 PMC3320611

[8] Haque E , Banik U , Monowar T , Anthony L , Adhikary AK . 2018. Correction: worldwide increased prevalence of human adenovirus type 3 (HAdV-3) respiratory infections is well correlated with heterogeneous hypervariable regions (HVRs) of hexon. PLoS One 13:e0196263. doi:10.1371/journal.pone.0196263 29590206 PMC5874027

[9] Crawford-Miksza L , Schnurr DP . 1996. Analysis of 15 adenovirus hexon proteins reveals the location and structure of seven hypervariable regions containing serotype-specific residues. J Virol 70:1836–1844. doi:10.1128/JVI.70.3.1836-1844.1996 8627708 PMC190011

[10] Yan H , Nguyen TA , Phan TG , Okitsu S , Li Y , Ushijima H . 2004. Development of RT-multiplex PCR assay for detection of adenovirus and group A and C rotaviruses in diarrheal fecal specimens from children in China. Kansenshogaku Zasshi 78:699–709. doi:10.11150/kansenshogakuzasshi1970.78.699 15478645

[11] Okada M , Ogawa T , Kubonoya H , Yoshizumi H , Shinozaki K . 2007. Detection and sequence-based typing of human adenoviruses using sensitive universal primer sets for the hexon gene. Arch Virol 152:1–9. doi:10.1007/s00705-006-0842-8 16957827

[12] Lee WJ , Kang C , Chung YS , Kim K . 2010. Molecular classification of human adenovirus type 7 isolated from acute respiratory disease outbreak (ARD) in Korea, 2005-2006. Osong Public Health Res Perspect 1:10–16. doi:10.1016/j.phrp.2010.12.005 24159434 PMC3766892

[13] Ylihärsilä M , Harju E , Arppe R , Hattara L , Hölsä J , Saviranta P , Soukka T , Waris M . 2013. Genotyping of clinically relevant human adenoviruses by array-in-well hybridization assay. Clin Microbiol Infect 19:551–557. doi:10.1111/j.1469-0691.2012.03926.x 22712766 PMC7129513

[14] International committee of Taxonomy of viruses. 2022. Available from: https://ictv.global/taxonomy

[15] Lynch JP , Kajon AE . 2016. Adenovirus: epidemiology, global spread of novel serotypes, and advances in treatment and prevention. Semin Respir Crit Care Med 37:586–602. doi:10.1055/s-0036-1584923 27486739 PMC7171713

[16] Meier JL . 2021. Viral acute gastroenteritis in special populations. Gastroenterol Clin North Am 50:305–322. doi:10.1016/j.gtc.2021.02.003 34024443

[17] Arowolo KO , Ayolabi CI , Lapinski B , Santos JS , Raboni SM . 2019. Epidemiology of enteric viruses in children with gastroenteritis in Ogun state, Nigeria. J Med Virol 91:1022–1029. doi:10.1002/jmv.25399 30636345

[18] Gaensbauer JT , Lamb M , Calvimontes DM , Asturias EJ , Kamidani S , Contreras-Roldan IL , Dominguez SR , Robinson CC , Zacarias A , Berman S , Melgar MA . 2019. Identification of enteropathogens by multiplex PCR among rural and urban guatemalan children with acute diarrhea. Am J Trop Med Hyg 101:534–540. doi:10.4269/ajtmh.18-0962 31392942 PMC6726947

[19] Kumthip K , Khamrin P , Ushijima H , Maneekarn N . 2019. Enteric and non-enteric adenoviruses associated with acute gastroenteritis in pediatric patients in Thailand, 2011 to 2017. PLoS One 14:e0220263. doi:10.1371/journal.pone.0220263 31369615 PMC6675392

[20] Pratte-Santos R , Miagostovich MP , Fumian TM , Maciel EL , Martins SA , Cassini ST , Keller R . 2019. High prevalence of enteric viruses associated with acute gastroenteritis in pediatric patients in a low-income area in vitória, southeastern Brazil. J Med Virol 91:744–750. doi:10.1002/jmv.25392 30614007

[21] Wu BS , Huang ZM , Weng YW , Chen FQ , Zhang YL , Lin WD , Yu TT . 2019. Prevalence and genotypes of rotavirus a and human adenovirus among hospitalized children with acute gastroenteritis in Fujian, China, 2009-2017. Biomed Environ Sci 32:210–214. doi:10.3967/bes2019.028 30987695

[22] Jones MS , Harrach B , Ganac RD , Gozum MMA , Dela Cruz WP , Riedel B , Pan C , Delwart EL , Schnurr DP . 2007. New adenovirus species found in a patient presenting with gastroenteritis. J Virol 81:5978–5984. doi:10.1128/JVI.02650-06 17360747 PMC1900323

[23] La Rosa G , Della Libera S , Petricca S , Iaconelli M , Donia D , Saccucci P , Cenko F , Xhelilaj G , Divizia M . 2015. Genetic diversity of human adenovirus in children with acute gastroenteritis, Albania, 2013-2015. Biomed Res Int 2015:142–912. doi:10.1155/2015/142912 PMC4538309 26339589

[24] Primo D , Pacheco GT , Timenetsky M do C , Luchs A . 2018. Surveillance and molecular characterization of human adenovirus in patients with acute gastroenteritis in the era of rotavirus vaccine, Brazil, 2012-2017. J Clin Virol 109:35–40. doi:10.1016/j.jcv.2018.10.010 30399502

[25] Afrad MH , Avzun T , Haque J , Haque W , Hossain ME , Rahman AR , Ahmed S , Faruque ASG , Rahman MZ , Rahman M . 2018. Detection of enteric- and non-enteric adenoviruses in gastroenteritis patients, Bangladesh, 2012-2015. J Med Virol 90:677–684. doi:10.1002/jmv.25008 29244212

[26] Gelaw A , Liebert UG . 2022. Molecular detection of enteric viruses in under-five children with diarrhea in debre tabor, Northwest Ethiopia. Infect Drug Resist 15:1981–1994. doi:10.2147/IDR.S364142 35480057 PMC9035461

[27] Hanieh S , Mahanty S , Gurruwiwi G , Kearns T , Dhurrkay R , Gondarra V , Shield J , Ryan N , Azzato F , Ballard SA , Orlando N , Nicholson S , Gibney K , Brimblecombe J , Page W , Harrison LC , Biggs B-A , Child Health and Nutrition Study team . 2021. Enteric pathogen infection and consequences for child growth in young aboriginal Australian children: a cross-sectional study. BMC Infect Dis 21:9. doi:10.1186/s12879-020-05685-1 33407180 PMC7788727

[28] Chhabra P , Payne DC , Szilagyi PG , Edwards KM , Staat MA , Shirley SH , Wikswo M , Nix WA , Lu X , Parashar UD , Vinjé J . 2013. Etiology of viral gastroenteritis in children <5 years of age in the United States, 2008-2009. J Infect Dis 208:790–800. doi:10.1093/infdis/jit254 23757337

[29] Wikswo ME , Kambhampati A , Shioda K , Walsh KA , Bowen A , Hall AJ , Centers for Disease Control and Prevention (CDC) . 2015. Outbreaks of acute gastroenteritis transmitted by person-to-person contact, environmental contamination, and unknown modes of transmission--United States, 2009-2013. MMWR Surveill Summ 64:1–16. doi:10.15585/mmwr.mm6412a1 26656915

[30] Akello JO , Kamgang R , Barbani MT , Suter-Riniker F , Leib SL , Ramette A . 2020. Epidemiology of human adenoviruses: a 20-year retrospective observational study in hospitalized patients in Bern, Switzerland. Clin Epidemiol 12:353–366. doi:10.2147/CLEP.S246352 32308491 PMC7147615

[31] De Francesco MA , Lorenzin G , Meini A , Schumacher RF , Caruso A . 2021. Nonenteric adenoviruses associated with gastroenteritis in hospitalized children. Microbiol Spectr 9:e0030021. doi:10.1128/Spectrum.00300-21 34319131 PMC8552676

[32] Kowada K , Takeuchi K , Hirano E , Toho M , Sada K . 2018. Development of a multiplex real-time PCR assay for detection of human enteric viruses other than norovirus using samples collected from gastroenteritis patients in Fukui Prefecture, Japan. J Med Virol 90:67–75. doi:10.1002/jmv.24926 28845896

[33] Fukuda Y , Tsugawa T , Nagaoka Y , Ishii A , Nawa T , Togashi A , Kunizaki J , Hirakawa S , Iida J , Tanaka T , Kizawa T , Yamamoto D , Takeuchi R , Sakai Y , Kikuchi M , Nagai K , Asakura H , Tanaka R , Yoshida M , Hamada R , Kawasaki Y . 2021. Surveillance in hospitalized children with infectious diseases in Japan: pre- and post-coronavirus disease 2019. J Infect Chemother 27:1639–1647. doi:10.1016/j.jiac.2021.07.024 34389224 PMC8332734

[34] Kim GR , Kim SH , Jeon GW , Shin JH . 2020. Prevalence of eleven infectious viruses causing diarrhea in Korea. Jpn J Infect Dis 73:427–430. doi:10.7883/yoken.JJID.2020.069 32475874

[35] Li W , Xiang W , Li C , Xu J , Zhou D , Shang S . 2020. Molecular epidemiology of rotavirus a and adenovirus among children with acute diarrhea in Hangzhou, China. Gut Pathog 12:19. doi:10.1186/s13099-020-00359-4 32313556 PMC7155314

[36] Wang L-P , Zhou S-X , Wang X , Lu Q-B , Shi L-S , Ren X , Zhang H-Y , Wang Y-F , Lin S-H , Zhang C-H , Geng M-J , Zhang X-A , Li J , Zhao S-W , Yi Z-G , Chen X , Yang Z-S , Meng L , Wang X-H , Liu Y-L , Cui A-L , Lai S-J , Liu M-Y , Zhu Y-L , Xu W-B , Chen Y , Wu J-G , Yuan Z-H , Li M-F , Huang L-Y , Li Z-J , Liu W , Fang L-Q , Jing H-Q , Hay SI , Gao GF , Yang W-Z , Chinese Centers for Disease Control and Prevention (CDC) Etiology of Diarrhea Surveillance Study Team . 2021. Etiological, epidemiological, and clinical features of acute diarrhea in China. Nat Commun 12:2464. doi:10.1038/s41467-021-22551-z 33927201 PMC8085116

[37] Kittigul L , Pombubpa K , Taweekate Y , Yeephoo T , Khamrin P , Ushijima H . 2009. Molecular characterization of rotaviruses, noroviruses, sapovirus, and adenoviruses in patients with acute gastroenteritis in Thailand. J Med Virol 81:345–353. doi:10.1002/jmv.21380 19107961

[38] Sriwanna P , Chieochansin T , Vuthitanachot C , Vuthitanachot V , Theamboonlers A , Poovorawan Y . 2013. Molecular characterization of human adenovirus infection in Thailand, 2009-2012. Virol J 10:193. doi:10.1186/1743-422X-10-193 23758792 PMC3693972

[39] Chaimongkol N , Khamrin P , Suantai B , Saikhreang W , Thongprachum A , Malasao R , Ukarapol N , Kongsricharoern T , Ushijima H , Maneekarn N . 2012. A wide variety of diarrhea viruses circulating in pediatric patients in Thailand. Clin Lab 58:117–123. 22372354

[40] Liu P , Xu M , Lu L , Ma A , Cao L , Su L , Dong N , Jia R , Zhu X , Xu J . 2022. The changing pattern of common respiratory and enteric viruses among outpatient children in Shanghai, China: two years of the COVID-19 pandemic. J Med Virol 94:4696–4703. doi:10.1002/jmv.27896 35641444 PMC9348017

[41] Ghaznavi C , Sakamoto H , Kawashima T , Horiuchi S , Ishikane M , Abe SK , Yoneoka D , Eguchi A , Tanoue Y , Hashizume M , Nomura S . 2022. Decreased incidence followed by comeback of pediatric infections during the COVID-19 pandemic in Japan. World J Pediatr 18:564–567. doi:10.1007/s12519-022-00575-9 35641696 PMC9154026

[42] Zhang J , Cao J , Ye Q . 2022. Nonpharmaceutical interventions against the COVID-19 pandemic significantly decreased the spread of enterovirus in children. J Med Virol 94:3581–3588. doi:10.1002/jmv.27806 35474224 PMC9088497

[43] Ismail AM , Cui T , Dommaraju K , Singh G , Dehghan S , Seto J , Shrivastava S , Fedorova NB , Gupta N , Stockwell TB , Halpin R , Madupu R , Heim A , Kajon AE , Romanowski EG , Kowalski RP , Malathi J , Therese KL , Madhavan HN , Zhang Q , Ferreyra LJ , Jones MS , Rajaiya J , Dyer DW , Chodosh J , Seto D . 2018. Author correction: genomic analysis of a large set of currently-and historically-important human adenovirus pathogens. Emerg Microbes Infect 7:208. doi:10.1038/s41426-018-0200-4 30532034 PMC6286714

[44] Lei Y , Zhuang Z , Liu Y , Tan Z , Gao X , Li X , Yang D . 2023. Whole genomic sequence analysis of human adenovirus species C shows frequent recombination in Tianjin, China. Viruses 15:1004. doi:10.3390/v15041004 37112985 PMC10142000

[45] Ji T , Li L , Li W , Zheng X , Ye X , Chen H , Zhou Q , Jia H , Chen B , Lin Z , Chen H , Huang S , Seto D , Chen L , Feng L . 2021. Emergence and characterization of a putative novel human adenovirus recombinant HAdV-C104 causing pneumonia in Southern China. Virus Evol 7:veab018. doi:10.1093/ve/veab018 33732504 PMC7953211

[46] Öner SZ , Kaleli İ , Demi R M , Mete E , Çalişkan A . 2022. Rotavirus and adenovirus prevalence in patients with acute viral gastroenteritis in Denizli, Turkey, 2017-2021. J Med Virol 94:3857–3862. doi:10.1002/jmv.27834 35510351

[47] Montasser KA , Youssef MI , Ghandour AA , Kamal M . 2022. Infection with adenovirus, rotavirus, and coinfection among hospitalized children with gastroenteritis in an Egyptian university hospital. J Med Virol 94:4950–4958. doi:10.1002/jmv.27935 35705322

[48] Olivares AIO , Leitão GAA , Pimenta YC , Cantelli CP , Fumian TM , Fialho AM , da Silva E Mouta S , Delgado IF , Nordgren J , Svensson L , Miagostovich MP , Leite JPG , de Moraes MTB . 2021. Epidemiology of enteric virus infections in children living in the Amazon region. Int J Infect Dis 108:494–502. doi:10.1016/j.ijid.2021.05.060 34052409

[49] Hassan F , Kanwar N , Harrison CJ , Halasa NB , Chappell JD , Englund JA , Klein EJ , Weinberg GA , Szilagyi PG , Moffatt ME , Oberste MS , Nix WA , Rogers S , Bowen MD , Vinjé J , Wikswo ME , Parashar UD , Payne DC , Selvarangan R . 2019. Viral etiology of acute gastroenteritis in <2-year-old US children in the post-rotavirus vaccine era. J Pediatric Infect Dis Soc 8:414–421. doi:10.1093/jpids/piy077 30184153

[50] Tran A , Talmud D , Lejeune B , Jovenin N , Renois F , Payan C , Leveque N , Andreoletti L . 2010. Prevalence of rotavirus, adenovirus, norovirus, and astrovirus infections and coinfections among hospitalized children in northern France. J Clin Microbiol 48:1943–1946. doi:10.1128/JCM.02181-09 20305010 PMC2863921

[51] Khamrin P , Okame M , Thongprachum A , Nantachit N , Nishimura S , Okitsu S , Maneekarn N , Ushijima H . 2011. A single-tube multiplex PCR for rapid detection in feces of 10 viruses causing diarrhea. J Virol Methods 173:390–393. doi:10.1016/j.jviromet.2011.02.012 21349292

[52] Kumar S , Stecher G , Li M , Knyaz C , Tamura K . 2018. MEGA X: molecular evolutionary genetics analysis across computing platforms. Mol Biol Evol 35:1547–1549. doi:10.1093/molbev/msy096 29722887 PMC5967553

